# Contextual Influences on the Success of Healthy Eating Policies and Practices in Australian Early Childhood Education Centres: A Qualitative Study with Directors

**DOI:** 10.3390/nu17162661

**Published:** 2025-08-17

**Authors:** Jacqueline Chan, Alexander Hyde-Page, Philayrath Phongsavan, David Raubenheimer, Margaret Allman-Farinelli

**Affiliations:** 1 Nutrition and Dietetics Group, Sydney Nursing School, Faculty of Medicine and Health, The University of Sydney, Sydney, NSW 2006, Australia; jacqueline.chan@sydney.edu.au; 2 Charles Perkins Centre, The University of Sydney, Sydney, NSW 2006, Australia; philayrath.phongsavan@sydney.edu.au (P.P.); david.raubenheimer@sydney.edu.au (D.R.); 3 Health Promotion Unit, Sydney Local Health District, Sydney, NSW 2037, Australia; alexander.hydepage@health.nsw.gov.au; 4 Prevention Research Collaboration, Sydney School of Public Health, Faculty of Medicine and Health, The University of Sydney, Sydney, NSW 2006, Australia; 5 School of Life and Environmental Sciences, The University of Sydney, Sydney, NSW 2006, Australia

**Keywords:** childcare, contextual factors, diet, health promotion, implementation, policy, preschool

## Abstract

Background/Objectives: Early childhood education and care is an ideal setting to promote healthy eating behaviours in young children. However, successful implementation and sustainment of healthy eating policies and practices remains a key challenge in the Australian early childhood education and care (ECEC) context. This study aimed to understand the contextual factors influencing early childhood education directors’ decisions to implement healthy eating policies and practices. Methods: Twelve directors from centre-based long day care centres in New South Wales, Australia, participated in semi-structured interviews. Interview data were analysed using reflexive thematic analysis and the Consolidated Framework of Implementation Research. Results: Directors (*n* = 12) described alignment with centre values and goals, compatibility with work infrastructure, local champions to lead implementation, and external partnerships with government support services as key facilitators. Directors identified a need for further support to address factors within the broader ECEC sector. Directors described a lack of external partnerships with the community, competing demands for available resources, unrealistic expectations from guidelines and parents, and inconsistent practices across settings as factors inhibiting implementation success. Conclusions: Implementation and sustainment of healthy eating policies and practices can be improved by strengthening parent and community partnerships, investment in the workforce, and a coordinated approach to the provision of support.

## 1. Introduction

Early childhood is characterised by rapid physical, social, emotional, and cognitive growth and is a critical period for ensuring children reach their developmental potential [1]. Optimal diet and nutrition in early childhood is fundamental for prevention of nutrient deficiencies [2], as well as motor and cognitive development [3] for ensuring school readiness [4]. The benefits of early exposure to good nutrition also extends beyond childhood and is associated with healthier diet quality [5] and reduced risk of cardiometabolic disease later in life [6].

Early childhood education and care (ECEC) centres have been identified as ideal settings for early childhood interventions to promote intake of healthy foods [7]. The ECEC setting provides a critical opportunity to reach large numbers of children and foster healthy eating behaviours. In Australia, almost 60% of children aged five years or under attended a centre-based day care and spent, on average, 27 h per week in care [8]. Children consume up to two-thirds of their recommended daily intake while in care, which can be influenced by many factors, including types and portion sizes of food served, meal service type, and feeding practices in the ECEC setting [9].

Recognising the important role that ECEC services plays in shaping healthy eating habits, most high-income countries have developed national or jurisdictional regulations and guidelines for the childcare sector, that recommend implementation of a number of healthy eating best practices [10]. Such practices include providing meals aligned with national dietary guidelines, offering nutrition education for children, establishing written nutrition policies, promoting supportive feeding practices, and creating healthy mealtime environments. However, the implementation of best practice standards that are consistent with such guidelines remains poor. For example, an assessment of ECECs in the United States found that centre-based services, on average, implemented only 7 out of 15 recommended nutrition practices [11]. In Australia, it has been reported that only 18% of centre-based services had all educators trained in nutrition [12]. Implementation factors remain a key challenge when facing sustained implementation of healthy eating policies and practices in the setting [13]. Adoption, implementation, and sustained delivery of nutrition interventions in the ECEC setting is complex, influenced by multiple factors and stakeholders [14]. It is thus important to understand how context, which describes the circumstances and factors surrounding implementation, can influence the desired outcomes [15,16]. Addressing contextual barriers and facilitators surrounding implementation can support the successful translation of research into sustained practice [17]. However, early childhood nutrition intervention studies rarely consider the broader environmental and policy context [18].

### Intervention and Policy Context in Australia

In Australia, the ECEC sector is regulated through the National Quality Framework, which assesses centres against seven quality standards areas [19]. The standards related to nutrition state that “Healthy eating […] are promoted and appropriate for each child” [19]. However, food provision guidelines, implementation support, and funding mechanisms to support centres to operationalise standards related to nutrition vary across states and territories [20]. Within the state of New South Wales (NSW), the Caring for Children framework was developed to provide practical guidelines on food, nutrition, and learning experiences [21]. Additionally, a statewide government-funded early childhood initiative, *Munch & Move*, has been implemented by Local Health District services to support the implementation of healthy eating and physical activity within ECEC centres [22]. Currently, only three out of eight states and territories in Australia have access to funded statewide nutrition programs.

Adequate implementation of recommended evidence-based healthy eating practices across Australia remains low, particularly for implementation of written nutrition policy, encouraging children to consume age-appropriate beverages, providing healthy eating education to children and families, and providing training and support to educators [23]. In the state of Victoria, a study found that, while government support services improved implementation of menu planning guidelines and menu quality, this did not always translate into menu compliance [24]. There remains an opportunity to better understand the factors influencing implementation of healthy eating policies and practices in the Australian ECEC setting to maximise potential benefits for young children.

In ECEC centres, directors play an important role in educational programming and organisational management, including managing staff, overseeing budget, and ensuring compliance with regulations [25]. To improve implementation of healthy eating in the ECEC setting, policies and practices need to reflect the challenges centre directors face in their roles and the context within which they are situated [26]. Qualitative research methods can generate meaningful data and provide an in-depth understanding of individuals’ unique experiences and context [27]. The aim of the results is not to generalise for broader populations but to provide rich descriptions to determine transferability of results in other contexts [28]. In this focal study of ECEC centres located in NSW, this offers useful insights to address the specific challenges faced in this context and inform implementation in other contexts.

Qualitative studies investigating the challenges in meeting dietary guidelines and achieving healthy food environments have previously been explored in the states of Queensland and Victoria; however, the existing studies focus on organisational factors within the ECEC setting context [24,29]. There remains a research gap in understanding the broader environmental and policy context and how these factors interact with the inner setting to provide understanding of a system for developing effective policy-level action. Specifically, this has not been explored in NSW, where 845,000 children attend centre-based care [19]. The aim of this study was to understand the experiences of directors regarding how contextual factors influence their decisions to implement healthy eating policies and practices in centre-based ECEC settings in NSW, Australia. 

## 2. Materials and Methods

This study follows the Standards for Reporting Qualitative Research (SRQR) reporting guideline [30]. This study was approved by the Sydney Local Health District Human Research Ethics Committee (Project Number X23-0379).

### 2.1. Study Design

This study used an interpretivist approach adopting a constructionist epistemological position, recognising that knowledge is not an objective reality but constructed by participant experiences and social context [31]. This approach is relevant for the exploration of the complex contextual factors that shape directors’ perspectives and decisions.

The authors bring a diverse range of expertise in health promotion research and evaluation, enabling the generation of rich insights through the co-construction of meaning from both health and early childhood education perspectives, ensuring relevance to practice.

### 2.2. Recruitment

Centre directors who were currently employed at long day care centres providing morning tea, lunch, and afternoon tea were eligible for the study. Directors were recruited from 783 centre-based services in Local Health Districts [8], located in areas with ethnically diverse populations. Purposive sampling was used to invite directors from centres engaged in the statewide *Munch & Move* initiative and hold knowledge on implementing healthy eating policies and practices. Health Promotion Service staff who support ECEC services to implement the *Munch & Move* initiative distributed recruitment material to directors through emails, e-newsletters, workshops, and a Facebook page. Data quality was reviewed during data collection and recruitment stopped when adequate information power was reached [32]. Given the specificity of the research question, use of the established CFIR framework to guide semi-structured interview questions, and sample of experienced and qualified directors (Table 1), we determined the sample size of 12 participants provided sufficient insight to generate an in-depth understanding of the factors influencing healthy eating policy and practice implementation [27,32]. Participant characteristics are presented in Table 1.

### 2.3. Data Collection

Semi-structured interviews were chosen for data collection to allow for the focused exploration of contextual factors within a limited timeframe, recognising the busy schedules of directors. A semi-structured interview guide was developed informed by the Consolidated Framework of Implementation Research (CFIR). The CFIR proposes a set of specific and detailed constructs in the inner setting domain to explain organisational factors and outer setting domain to explain the broader factors influenced by where the organisation is placed in the community, political, and economic environments [33]. The CFIR has previously been used in the childcare and school setting to assess barriers and facilitators of healthy eating and obesity prevention programs [34,35,36].

The semi-structured interview guide consisted of seven open-ended questions to allow for open exploration of contextual factors, as well as prompts to explore factors outlined by CFIR (Table 2). The guide was pilot tested with an ECEC director not included in this sample before use to ensure questions were appropriate and relevant.

All interviews were conducted by the first author (J.C.) at the ECEC centre where the directors were employed. Participants provided informed written and verbal consent prior to participation. The interviews were audio-recorded using Zoom software (version 6.4.12). Audio recordings were transcribed verbatim in Microsoft Word following the interviews. Interviews were conducted between December 2023 and April 2024. The duration of the interviews ranged from 20 to 67 min, with a mean of 35 min. Participants were provided with an AUD 30 gift voucher as reimbursement for their time.

### 2.4. Data Analysis

Reflexive thematic analysis is well suited to a constructivist perspective, as it emphasises the role of the researcher in the co-construction of knowledge [37]. The flexibility of this approach allowed authors to use CFIR to deductively map contextual factors while inductively engaging with the data to generate themes relevant to the ECEC context. This allows for a structured approach to examine the inner and outer setting factors relevant to the study aim while also capturing how participants made sense of their experiences. The six-phase process, as described by Braun and Clarke, was used [38]. One data coder (J.C.) read through all the transcripts to become familiar with the data. Transcripts were first inductively coded by J.C. to allow codes and themes to emerge from the data. This was followed by a second level of coding guided by CFIR to articulate specific concepts. The codes from two transcripts were reviewed by a second coder (M.A.-F.). Discussion between the two data coders allowed for refinement and the addition of codes from a different perspective to provide a more nuanced interpretation of the data. One data coder (J.C.) then analysed the codes to generate candidate themes and sub-themes from patterns of meaning in the data. Candidate themes were refined by reviewing themes against coded data and re-reading the entire data set to ensure codes and themes fit. The final themes were organised and presented using the CFIR’s inner and outer setting domains to support the interpretation in line with the study’s aims and to highlight contextual factors influencing implementation. Themes were reviewed to define the central organising concepts and named. Data were managed and analysed using NVivo 14.

## 3. Results

Directors from twelve long day care centres participated in semi-structured interviews. Five key themes were developed relating to how directors described the influence of contextual factors on decisions to implement healthy eating policies and practices (Table 3).

### 3.1. Theme 1: Inner Setting Factors

#### 3.1.1. Healthy Eating “Just Happens”

Directors described shared values and goals around supporting children’s needs and described providing healthy nutritious food as an “important part of our existence” (Participant 39). Directors recognised that many families were poor and may not be able to provide nutritious meals at home. As children were spending long hours in childcare, directors expressed fulfilment in providing nutritious food.

Directors’ descriptions of their role in implementing healthy eating learning experiences were less consistent. Some explained that healthy eating was included as part of the centre’s programmed curriculum alongside science, maths, and literacy. For others, healthy eating learning experiences were seen as an important extracurricular, that is, “part of” but not a core aspect of the curriculum.

“I would probably say that educators probably think more the core is more like social and emotional and things like that”

(Participant 24, non-profit centre)

Directors described leveraging other learning opportunities to integrate healthy eating. For instance, participant 14 described implementing excursions to native gardens as an opportunity for nutrition education, to support the indigenous community, and talk about “caring for the earth as well”. Directors also described other opportunities to integrate healthy eating while “also teaching them science” (Participant 12) and developing children’s sensory perception and motor skills. Many described school readiness programs as a good opportunity to include healthy eating through lunchbox activities.

Directors often described healthy eating practices occurring organically and as needed. Directors felt that there were many opportunities for healthy eating learning experiences through discussions at mealtimes and daily conversations.

“I wouldn’t say there’s a set time. There’s obviously daily spontaneous conversations, but if it does come up […] we’ll plan a follow up experience.”

(Participant 19, for-profit centre)

Directors expressed that allowing staff to plan healthy eating learning experiences based on their interest increased staff motivation and facilitated implementation. Healthy eating learning experiences were also prompted by cultural celebrations and food-related events throughout the year or planned in response to educator, child, or family interest. Participant 24 compared the implementation of practices such as talking about healthy eating as something that “happens a lot” and “just happens” compared to physical activity, which needed to be “planned intentional like things that we focus on” to fulfill specific fundamental movement skill outcomes.

Directors expressed they were content with the way healthy eating policies and practices were currently being implemented and agreed informal practices were working well within their centres. When asked if any changes to internal practices were required, directors often expressed little need for change. Directors noted that, because their nutrition policy was “not very big” (Participant 24) and nutrition experiences occurred sporadically, they did not feel there were many restrictions or barriers to implementation.

“So obviously we wouldn’t be talking about nutritional planning, nutrition experiences every day or even so every week, it might just be sporadically, you know, throughout the year. So I don’t feel that there’s very many barriers.”

(Participant 19, for-profit centre)

#### 3.1.2. Navigating Competing Priorities

Directors articulated many complexities and competing priorities around food provision. Directors frequently commented on food allergies and anaphylaxis, suggesting this was at the forefront of planning and practice. Directors described challenges meeting nutrition guidelines while managing allergies. For example, participant 3 commented “the nutritional value of an egg is gone for the whole service, so it’s very hard”. Providing opportunities for children to make their own choices around food was frequently noted as an important value that directors described as a challenge to managing fussy eating. Other competing priorities described included minimising food wastage, ensuring quality of food, and food safety and hygiene.

Directors frequently commented on the competing demands for available financial resources, including the increasing costs of food, mandatory staff training requirements, and staff wages.

“So the cost of living is rising too much, so that is one of the things that we are getting conscious of […] We did have children with soy allergies, so definitely there is another type of bread for them. So again, it’s cost, cost, cost added to it.”

(Participant 1, for-profit centre)

“There’s big pressure to bring costs down and wages are going up and there’s staff in crisis at the moment […] People are using staff from agencies which are costing a lot […] and I think they’re cutting corners a lot in terms of healthy eating and nutrition.”

(Participant 39, non-profit centre)

Directors from for-profit centres reflected that “it always comes down to money” (participant 25), and some explained costs were passed onto families through increased fees. Others applied for external grants, which enabled centres to prioritise healthy eating and support the implementation of interventions. However, securing external grant funding was challenging, as opportunities were scarce and a lot of paperwork was required.

Directors acknowledged staffing shortages across the ECEC industry; however, most directors described low staff turnover and dependable staff within their own centres. Directors noted when staff time was limited, it was usually allocated to primary responsibilities, including managing children for educators and food preparation for cooks, rather than the delivery of healthy eating practices. Some centres decided to use catering services to overcome these challenges and ensure children were provided with fresh and nutritious food even when staff were not available.

#### 3.1.3. Supportive Work Infrastructure

Directors who were confident in staff and their own capability often reported available resources. Directors described company support, including in-house dietitians, company-wide training for staff, and funding for mandatory training. For those part of a larger company, there were internal networking opportunities for directors and staff to support each other, such as WhatsApp group chats. Some also noted flexibility within the budget for food and flexibility to implement policy and practices according to local conditions. Some directors noted that the organisation of tasks and responsibilities within their team enabled learning experiences to be planned if an opportunity “pops up” (Participant 48). For example, chefs providing recipes or organising ingredients for educators to deliver cooking experiences.

For some, the ability to rely on informal practices was facilitated by local champions. Directors described that implementation of healthy eating practices was driven by passionate staff committed to providing high quality food and new learning experiences.

“You couldn’t replicate this everywhere. I think if you had high turnover and just say she was to retire and the person you replaced her with wasn’t as passionate it could be a completely different.”

(Participant 39, non-profit centre)

Some directors noted staff were adaptable and flexible to fit changing needs and processes in place, such as regular team feedback to resolve issues. Some cited that their experience in the industry was a key facilitator to overcoming challenges, reflecting that “the only barrier is you know how much you push yourself” (Participant 14).

### 3.2. Outer Setting Factors

#### 3.2.1. Satisfactory Implementation Support

Directors felt like they had access to support from a statewide government support service, *Munch & Move*, to implement policy or practices according to guidelines. Directors cited *Munch & Move* as the primary resource for healthy eating, including workshops for staff, written materials for parents, and resources to support staff to deliver learning experiences to children. Directors felt most supported for menu planning, noting the approval process and certificate as an incentive. Some directors noted that the government support service was more than just a resource for information, they felt that the relationship and trust with health promotion officers was a facilitator for implementation. One director expressed that they felt more comfortable reaching out to *Munch & Move* than approaching their company head office for support.

“So it’s not just sending our stuff in the mail. Like we have a great connection with them, so it’s really good trust.”

(Participant 14, for-profit centre)

#### 3.2.2. Regulations vs. Reality

Some directors expressed that external quality standards and guidelines related to food and nutrition were necessary to safeguard the ECEC industry and ensure high quality care. For others, policies and guidelines were an obligation. Directors stated they had a duty of care to children and families to follow them and “tick all of those boxes” (Participant 19).

External nutrition policies and guidelines were seen as unrealistic. Some directors noted that the requirements for red meat did not align with family preferences and some noted that the requirements for vegetables were excessive, leading to food wastage and higher food costs. Some directors also felt that the requirements for healthier alternatives were too costly.

“And we’ve tried to take this suggestion on board, and often it leads to a lot of food wastage […] Even though we sat with children and role model and involve them in the process.”

(Participant 12, non-profit centre)

#### 3.2.3. Parent vs. Partnership

Directors expressed concerns about the differences in practices between the home and the ECEC food environment and how this could potentially “confuse children” (Participant 16). Directors described discrepancies being attributed to a lack of parental nutrition knowledge, differences in cultural values, or, for others, directors noted families were poor and tight on money.

Directors expressed communication with parents was important for building partnerships between ECEC and families. Directors also noted the importance and responsibility of ECECs to support families with healthy eating practices at home. This included documenting healthy eating learning experiences and sharing with parents, organising parent evenings, distributing newsletters and information sheets, and sharing links to workshops. However, some directors reflected there was little engagement from parents and, as such, felt limited in what they could do to support families.

“Yes, but we need to honour whatever the parents decide. And that is a bit of a barrier because we can try and talk to them, but at the end of the day they can make their choices for their child.”

(Participant 22, for-profit centre)

Directors also described facing parental pressure to manage fussy eating behaviours and cater to family preferences. Some directors felt that they had to honour parents’ wishes despite going against recommendations and guidelines, creating tension between trying to please parents and meeting policies and guidelines.

“We had a complaint where they said nothing was homemade and then we changed the menu with all homemade and then we check […] with *Munch & Move*, they said you can’t have everything baked […] I’m like, Oh my God, it’s hard to like please everybody […] but you know, we’re not the Hilton and we’re not going to be providing like, gourmet every day.”

(Participant 3, for-profit centre)

Directors expressed the need for more information and resources to support families, particularly for first time parents. Some suggested more support is needed to implement “hands-on” healthy eating interventions that involve the home environment, such as an app or workshops for families. Directors noted there was an existing relationship with the local council and reflected on the successful implementation of environmental sustainability programs. Following the success of these interventions, directors recommended that aspects of healthy eating and nutrition should be incorporated.

#### 3.2.4. Fragmented Systems of Support

Systems to support implementation of healthy eating policy and practice were seen as inadequate to support the ECEC sector. Directors expressed that they were interested in more information and new ideas for how to implement healthy eating policies and practices, but noted it was challenging to stay up to date with available resources. Directors described resources as scattered and recommended a directory or service to support directors in locating current resources. Directors noted that they often rely on their own research and internal support to stay up to date with new information, resources, and funding opportunities.

“It’s very time consuming […] it’s Googling, it’s looking for them […] But for something like that I wouldn’t even know where to look. So, I have support with the parent management committee to sometimes help me lock down grants.”

(Participant 39, non-profit centre)

Directors felt that the National Quality Standards were “not extremely specific like NSW Health” and centres could “interpret them the way that they wish” (Participant 19). However, there was tension between the ideal of implementing more specific guidelines for consistency across the ECEC sector and the reality of implementation. Directors reflected on the lack of external partnerships and connections with the community. Directors also expressed concerns that new staff had inadequate nutrition education during their early childhood educator training. Directors described limited ongoing professional development opportunities and recommended funding for online modules or workshops to support staff. Furthermore, directors felt there was a lack of funding and policy to support the challenges faced by the ECEC industry, such as staff shortages.

“We already have enough guidelines, enough regulations, we just don’t have the funding, or the education for it. So, yeah, those are the big issues because like, yes, you can add the regulation, but that’s not going to change anything.”

(Participant 22, for-profit centre)

## 4. Discussion

The current study identified the contextual factors influencing ECEC directors’ decisions to implement healthy eating policies and practices. Directors articulating implementation success described supportive inner setting factors, including a strong culture of shared values and goals to support children’s growth and development and work infrastructure to organise tasks and responsibilities for staff, as well as having a local champion to lead the implementation. However, directors noted that competing demands on resources, such as centre funding and staff time, were a key barrier. Directors also acknowledged outer setting partnerships with Local Health District services to support the implementation of healthy eating guidelines. Directors often described outer setting factors inhibiting implementation success, including lack of external partnerships with the community, unrealistic guidelines, parental pressure, and inconsistent practices at home. Directors expressed that local conditions were not supportive, citing workforce and funding issues across the ECEC sector. The sustained implementation of healthy eating policy and practices depends on key outer contextual factors, including having a policy or guideline from a governing body, alignment with the broader community, such as parents, and external partnerships [39].

Within the inner setting, directors described having flexibility in their food budgets and the ability to pass on costs to families through fee increases, enabling the provision of nutritious meals. Although directors described a complex environment of competing demands for available resources that impacts on the implementation of healthy eating policies and practices, participants noted a supportive work infrastructure, such as provider support for in-house dietitians and training, which enables healthy eating learning experiences to “just happen”. These experiences may be specific to centres in advantaged areas represented in this sample and may not reflect those of centres in disadvantaged areas. In contrast, centres located in disadvantaged areas are less likely to provide meals, and when meals are offered, their provision is not associated with fee increases, as parents’ ability to pay constrains pricing, thereby limiting food budgets and flexibility [40]. These centres may also experience greater job demands to meet the needs of children and families experiencing disadvantages [41]. A previous study found that implementation was less likely if healthy eating was perceived as less important compared to other priorities [36]. Opportunities to use programs to meet multiple curriculum requirements and the ability for these programs to “fit” within existing workflows and systems can facilitate the implementation and sustainability of nutrition and physical activity interventions in the ECEC setting [42]. Given competing demands, strengthening connections between healthy eating and the learning outcomes at the forefront of planning and practice through multisectorial partnerships may better align with director priorities and support implementation.

Networks and partnerships with external organisations have been identified as important facilitators to supplement available resources and provide partners for the implementation of healthy eating activities [36,42]. In the current study, partnership with a statewide government service and targeted healthy eating support were key facilitators, aligning with the existing literature and highlighting the importance of implementation support in enhancing policy and practice adoption in ECEC settings [13]. However, directors expressed a need for a coordinated system to access information and more community partnerships, suggesting greater input from the local council. Local councils are well placed to provide support to improve implementation of healthy eating interventions in ECECs given their role in providing goods and services to their communities, local planning, and implementing local food system policies [43]. Multisectoral partnerships between local council, local health services, and ECEC regulatory authorities could provide a more coordinated approach to support directors in their role to implement healthy eating policies and practices [10].

Previous research suggests that ECEC centres are more likely to implement healthy eating interventions when they have parental support [36]. However, similar to the findings of this study, others report that parental disengagement and inconsistencies between home and ECEC settings are common challenges [29,44]. Effective workflow designs that promote regular communication and collaboration with parents are key to addressing disengagement [42,45]. Inclusion of resources to support staff in communicating and collaborating with parents through existing ECEC nutrition support services and programs can improve parent partnerships [46]. To support this, existing programs like *Munch & Move* (NSW) and Healthy Eating Advisory Service (Victoria) could be expanded to include family engagement components. Directors in this study recommended home-based learning activities, workshops, and a digital tool such as an app for families. Policy incentives could encourage ECEC services to promote parent engagement and improve healthy behaviours across home and ECEC settings. For example, in the United States, the Child and Adult Care Food Program (CACFP) is a federally funded initiative that ties funding for meal subsidies with standards that require ECEC services to communicate with parents about child nutrition [47].

Consistent with previous research, directors described many informal opportunities to implement healthy eating practices [29]. However, studies conducted in NSW and Victoria, Australia, suggest current healthy eating environments and practices do not align with best practice guidelines [12,48]. Directors may not always express the need for change if not aware of how and why healthy eating practices should be implemented [44]. Additionally, weak nutrition policies that are not tailored to centres and lack adequate detail may result in poor implementation of healthy eating practices [29,48]. It would be in the best interests of children if there was a national agreement to set clear objectives regarding food provision and healthy food environment outcomes to ensure consistent interpretation across the ECEC sector. However, the findings from this study suggest further investment in resourcing is needed to support the implementation of stronger nutrition standards. For example, in the United States, ECEC centres receiving CACFP subsidies for serving healthy meals reported fewer barriers to implementing new healthy eating policies [49]. Similar to CACFP, introducing a funding model in Australia that requires centres to meet nutrition guidelines as a condition of receiving support may improve compliance with nutrition standards and promote healthier food environments.

In this study, state guidelines were perceived as unrealistic by the directors, failing to acknowledge the competing demands and complexities of the setting. While many directors were “lucky” with their staff, directors expressed the need to fix underlying issues with the ECEC workforce, such as poor staff retention and insufficient nutrition knowledge, before making changes to the rules and regulations. Staff shortages and turnover in the Australian ECEC context have been well described and attributed to high demand, low staff wages, poor work conditions, and lack of recognition [41]. Dependable staff have been found to be important facilitators in this study and others [42], emphasising the need for long-term investments from national and subnational budgets to ensure a skilled ECEC workforce that meets the level of demand [10]. Nutrition training should be standardised and mandated for all ECEC staff by embedding comprehensive nutrition and responsive feeding content in early childhood education qualifications and providing ECEC specific nutrition training for cooks.

Despite the “workforce challenges”, staffing was rarely identified as a barrier in this study, possibly reflecting that a skewed sample was recruited. This may be because directors experiencing resource constraints likely lack the time or capacity to participate. Additionally, while recruitment included directors from socio-economically diverse areas, all participants who volunteered to participate were from advantaged areas, which may limit transferability of the findings to ECEC services in disadvantaged communities. These settings are often more likely to experience implementation challenges such as resource constraints [40]. Further research is needed in disadvantaged communities to better understand the contextual barriers and enablers that shape implementation in these settings. Although all participants recruited were female, this reflects the gender composition of the Australian workforce in this sector. In 2024, the ECEC National Workforce Census reported that 91% of the ECEC workforce was female [50]. Although the findings were similar to those described in recent studies from other jurisdictions within Australia [24,29], we acknowledge this sample may not capture all experiences. However, the findings offer insights from directors who are engaged with and interested in nutrition and food practices, which can provide valuable information that those indifferent to the topic may overlook. This study addresses important gaps in the literature, as the majority of studies examining barriers and facilitators to the implementation of nutrition in the ECEC setting focus on describing the inner setting factors and lack theoretical guidance, with only 28% of studies using theories, models, or frameworks to guide evaluations [42]. A strength of this study is that it used both an inductive approach to allow for engagement with the rich data and a deductive approach informed by the CFIR to systematically identify inner and outer setting factors. This approach generates practical insights to inform the development of policy and practice recommendations that more accurately reflect the real-world challenges faced by ECEC settings. Further research to understand the unique outer contextual factors experienced by disadvantaged populations across Australia and other ECEC centre types such as family day care is recommended to inform policy and practice.

## 5. Conclusions

This study provides a greater understanding of contextual factors influencing directors’ decisions to implement healthy eating policies and practices in the ECEC setting. Inner setting contextual factors, including alignment of goals and values, fit within work infrastructure, and implementation leads, were identified as facilitators. External partnership with government support services was identified as an enabler within the context of NSW, Australia. The study identified the need for further support to navigate the outer setting contextual factors impeding implementation. The ECEC setting may benefit from strengthening community partnerships with the local council, expanding government-funded implementation support to include family engagement components, investment in the workforce through standardised and mandated nutrition training for all staff, and provision of conditional funding support that encourages ECEC services to meet nutrition standards. A combination of “upstream” national policy actions to improve the outer contextual factors and “downstream” approaches that focus on the inner setting factors is recommended.

## Figures and Tables

**Table 1:** Demographic characteristics of participating directors and centre characteristics (*n* = 12).

Participant Characteristics	n (%)
Gender	
Female	12 (100)
Age	
18–29 years	2 (17)
30–39 years	6 (50)
40–49 years	3 (25)
50–59 years	1 (8)
Highest level of education	
Degree (Bachelor, Masters, Doctorate)	10 (83)
Graduate or Advanced Diploma	2 (17)
Median years of experience in ECEC (Range)	13.5 (5–28)
Country of birth	
Australia	6 (50)
Other ^†^	6 (50)
**Centre characteristics**	**n (%)**
Number of children	
Range (n)	35–98
Average (n)	62
Service provider type	
For-profit	9 (75)
Non-profit	3 (25)
Food preparation	
All meals cooked on-site	9 (75)
All meals are outsourced	1 (8)
Mixed	2 (17)
Socio-Economic Status (SEIFA 2021) ^‡^	
Most disadvantaged	0 (0)
Most advantaged	12 (100)

^†^ China, Italy, Ireland, Mauritius, Pakistan, and the Philippines. ^‡^ ISRAD Quintile: Index of Relative Socio-economic Advantage and Disadvantage (ISRAD) provides a measure of advantage and disadvantage based on economic and social conditions, such as income and occupation, of people and households within a geographical area. A high score indicates a relative lack of disadvantage and greater advantage. Most disadvantaged = quintiles 1–3. Most advantaged = quintiles 4–5.

**Table 2:** Interview guide outlining the interview questions and associated Consolidated Framework for Implementation Research domain.

CFIR Domain Objective	Interview Questions
Context To identify interventions currently being implemented	Can you tell me about any food or nutrition policies/guidelines, practices or programmes that are currently provided at your service? a What do you like? b What do you dislike?
Innovation To identify the components of the innovation that enable and/or inhibit implementation and sustainment of nutrition interventions.	2. What features would you need to continue, improve, or discontinue? Prompts: - What would you change and why? What would you keep and why?
Inner setting To identify the inner setting organisational barriers and enablers to implementation and sustainment of nutrition interventions.	3. How does providing a nutrition policy, practice, or programme fit in with the priorities of your service? Prompts: - What are the values/beliefs around nutrition 4. Tell me about the resources available in your service for prioritising nutrition policy, practices and programmes. Prompts: - How does staffing influence how your service prioritises nutrition? - How does cost/budget influence your service to prioritise nutrition? - What are the workflows/processes that influence how you prioritise nutrition at your service. - What are the communication strategies between management, educators, cooks, and families at your service to support healthy eating.? 5. What resources do you need to continue to provide it?
Outer setting To identify the outer setting barriers and enablers to implementation and sustainment of nutrition interventions.	6. Tell me about the factors outside the control of your service that influence your decision to provide a nutrition policy, practice, or programme? Prompts: - How is your decision influenced by external funding? (e.g., subsidies, re-imbursements, grants from government or non-government organisations or lack thereof) - How is your decision influenced by external policies or guidelines? - How is your decision influenced by national quality benchmarks/accreditation standards? - How is your decision influenced by external entities (e.g., professional networks, government or academic organisations, community partners)? 7. What external support do you have/would you need to continue to provide it?

**Table 3:** Overview of the themes describing contextual factors influencing directors’ decisions to implement healthy eating policies and practices in centre-based early childhood education and care services.

Themes	Sub-Themes	Overview
Inner Setting factors	1.1. Healthy eating “just happens” 1.2. Navigating competing priorities 1.3. Supportive work infrastructure	Inner Setting factors captures the barriers and enablers at the organisational level within ECEC services. Directors described implementation of healthy eating experiences was described as something that “just happens”. Competing priorities for available resources such as funding and staff time was described as a barrier. Supportive work infrastructure was described as a facilitator.
2. Outer Setting factors	2.1. Satisfactory implementation support 2.2. Regulations vs. reality 2.3. Parent vs. partnership 2.4. Fragmented systems of support	Outer Setting factors captures the barriers and enablers at the system level across the ECEC sector. In the context of NSW, *Munch & Move* was identified as a key facilitator. External pressure from nutrition guidelines, the local attitudes of parents, and local funding conditions were identified as barriers.

## Data Availability

The data presented in this study are available on request from the corresponding author due to ethical restrictions.

## Abbreviations

The following abbreviations are used in this manuscript:

CFIR	Consolidated Framework for Implementation Research
ECEC	Early Childhood Education and Care
NSW	New South Wales
